# On the interplay between time and space perception in discontinuous stimulus displays

**DOI:** 10.3758/s13414-023-02678-5

**Published:** 2023-02-24

**Authors:** Wladimir Kirsch

**Affiliations:** Institut für Psychologie III der Universität Würzburg, Röntgenring 11, D-97070 Würzburg, Germany

**Keywords:** Kappa effect, Tau effect, Space-time dependency

## Abstract

The present study examined whether and how the mutual perceptual biases of temporal and spatial information, known as the kappa and the tau effects, depend on the duration and spatial extent of sensory stimulation as well as on the magnitude of spatio-temporal discrepancy. Three small circles were presented in succession at different spatial positions. The time points of presentation and the spatial position of the second circle systematically varied. Participants judged either whether the temporal interval between the first and the second circle was longer than the interval between the second and the third circle (Experiment 1) or whether the spatial distance between the first and the second circle was larger than the distance between the second and the third circle (Experiment 2), or both in separate blocks of trials (Experiment 3). The impact of spatial information on temporal perception (i.e., the kappa effect) increased with velocity of motion presumably imputed by the participants to the static displays and decreased with spatio-temporal discrepancy. No inverse biases (i.e., no tau effects) were observed. These results are considered as an indication that integration of spatial and temporal signals follow the same basic principles as multisensory integration of redundant signals, such as those from vision and touch.

## Introduction

Space and time are basic dimensions of human experience that are usually studied in separate research domains. It is well known, however, that there are mutual interactions between these dimensions, known as the “kappa” (Cohen et al., 1953) and “tau” effects (Helson, 1930). In a standard paradigm, three transient stimuli (e.g., light flashes) are presented in succession at different spatial positions. There are thus two spatial and two temporal intervals defined by the stimulus presentation. When asked to compare the temporal intervals participants’ judgments systematically vary as a function of the spatial intervals: a spatially larger interval is judged as temporally longer (kappa effect). And vice versa, when asked to compare the spatial intervals participants’ judgments systematically vary as a function of the temporal intervals: a temporally longer interval is judged as spatially larger (tau effect). Both effects appear to be robust phenomena as they were demonstrated with diverse methods and under diverse stimulus conditions (see, e.g., Helson & King, 1931, and Scholz, 1924, for the studies on the tau effect; Abe, 1935, Cohen et al., 1953, and Price-Williams, 1954, for the studies on the kappa effect; and Jones & Huang, 1982, for a review).

A common explanation for the kappa and tau effects rests on the assumption that observer imputes motion to the static display and bases her/his judgment upon the physical relationship between distance (s), time (t) and mean velocity (v) (v = s/t; Collyer, 1977; Huang & Jones, 1982; Jones & Huang, 1982; Price-Williams, 1954). More precisely, observer assumes that a spatial-temporal stimulus sequence reflects a motion of a single stimulus with constant velocity (“constant velocity hypothesis,” e.g., Jones & Huang, 1982). An increase in a spatial interval between two stimuli should hence lead to an increase in the temporal interval between them, and vice versa, an increase in a temporal interval should lead to an increase in the spatial interval (because velocity is assumed to be constant, as mentioned). In other words, the perceived time and distance (i.e., the kappa and tau effects, respectively) are the result of the actual time and distance, and of the expected time and distance. The expected values are derived from the known distance and velocity. Algebraically, the actual and expected values are averaged taking the relative weights of these values into account.

More recently, a similar idea was treated within a Bayesian approach aiming at explanation of spatiotemporal illusions including the tau effect in the somatosensory system (Goldreich, 2007; Goldreich & Tong, 2013). This approach suggests, in essence, that observers generally expect slow velocities when confronted with a series of successive stimuli presented at different locations. Combining this “prior” with the actual spatiotemporal information (i.e., “likelihood”) in a statistically optimal way, i.e., taking the relative precision of prior and likelihood into account, should naturally reveal tau- and kappa-like effects (i.e., “posterior”) not only in the somatosensory modality. The constant velocity hypothesis can also be treated as a Bayesian model, in which the time and distance expected in a given trial (and derived according to v = s/t; see above) represent a “prior” (see also Chen et al., 2016). Thus, both approaches commonly propose that kappa and tau effects arise when sensory input and expectations about this input are combined in perception. Both also presume that constant speed is computed based on the spatial and temporal information of the successive target presentations (see also Goldreich, 2007). The main difference thus relates to whether slow or constant velocity is expected (i.e., constant velocity vs. slow velocity prior; see also Chen et al., 2016).

Having a considerable explanatory value, these approaches to the spatial-temporal biases in perception are limited. The constant velocity hypothesis, for example, does not explain and thus does not allow us to predict how the relative weights are assigned (see also Chen et al., 2016; Goldreich, 2007). The theoretical implications of the low-velocity expectation hypothesis are debatable. In particular, always predicting no movement in the environment should lead to a systematic underestimation of velocities of all moving objects. This could substantially endanger the organism and thus might be not a reasonable strategy from an evolutional point of view (for this argument and other points of criticism, see also Merz et al., 2022). Moreover, researchers sometimes do not observe the usual kappa and tau effects, but rather no effects or effects of the opposite directions (e.g., Collyer, 1977; Huang & Jones, 1982; Roy et al., 2011). Also, it has been reported that the kappa and tau effects emerge when the attended dimension is constant and the other dimension is variable (i.e., when the spatial intervals are the same and the temporal intervals vary in case of spatial judgments, and when the temporal intervals are the same and the spatial intervals vary in case of temporal judgments) but not when the attended dimension is variable and the other dimension is constant (i.e., when the spatial intervals are the same and the temporal intervals vary in case of temporal judgments and when the temporal intervals are the same and the spatial intervals vary in case of spatial judgments; Sarrazin et al., 2004; Sarrazin et al., 2007). Such observations are difficult to reconcile with any of the previous approaches.

**Fig. 1:**
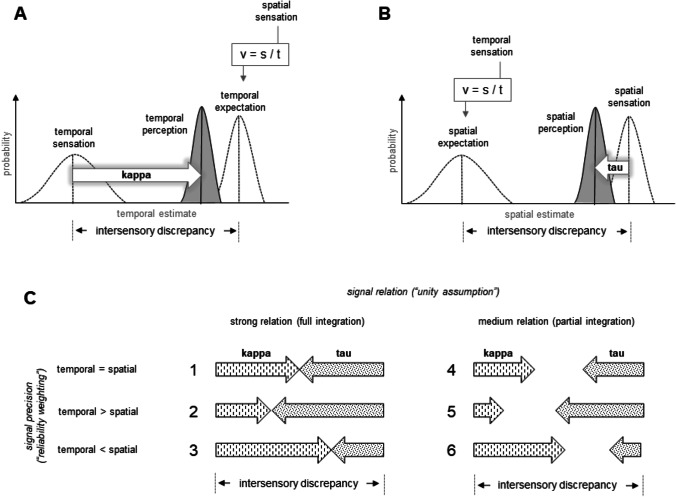
Changes in temporal and spatial perception (i.e., kappa and tau effects) as a function of relative signal precision and inferred signal relation. Fictitious likelihood functions reflecting the probability distribution of temporal and spatial judgments are illustrated in (**A** and **B**) (i.e., the x-axis represents different magnitudes of temporal and spatial intervals, while the y-axis reflects how likely these perceptions are). The filled functions represent perceptual judgments based on integration of actual and expected sensations (represented by unfilled functions). The peaks indicate the most likely perceptions, whereas the width of such a function indicates how precise or reliable the underlying signal is (the wider the less precise). How the magnitude of the kappa and tau effects changes depending on signal precision and signal relation is shown in (**C**)

I feel that the kappa and the tau effects as well as many related biases can better be understood considering some known regularities of sensory integration of redundant signals. The basic idea is that both temporal and spatial signals inform about the same “event” (i.e., about an apparently moving object), like vision and touch inform about the grasped object we are looking at. Such redundant cues are usually assumed to be optimally combined to decrease the variance in the perception of the event. The integrated percept is a weighted average of individual signals and the weights represent the relative signal precision (or reliability): a more precise signal receives a larger weight (“reliability weighting”; e.g., Ernst & Bülthoff, 2004; Ernst & Banks, 2002; Welch & Warren, 1980). Another known regularity suggests that the extent to which the signals are integrated depends on how strong the perceived signal relation is (“unity assumption”; e.g., Chen & Spence, 2017; Körding et al., 2007; Roach et al., 2006; Shams & Beierholm, 2010). The stronger the signal relation inferred by the observer is the stronger is sensory integration. Accordingly, the magnitude of integration can vary from “full integration” (also called “fusion”) to “segregation” including “partial” integration. Crucially, if an intersensory discrepancy^1^ is introduced, this multisensory approach predicts mutual perceptual biases such as the kappa and tau effects (see Fig. 1).

In general, thus, temporal and spatial signals that commonly inform about a spatiotemporal event can be integrated to a varying degree by taking the precision of each signal into account. If there is a discrepancy between these signals, their integration results in mutual perceptual biases (i.e., in kappa and tau effects) because, in essence, integration means signal averaging. The strength of integration (unity assumption) and the relative signal precision (reliability weighting) commonly determine the magnitude of these biases. For example, assume temporal and spatial signals are equally reliable and the observer fully integrates them because they are strongly correlated (i.e., each signal receives a weight of 0.5). In this case, the magnitudes of the kappa and the tau effects would correspond to the magnitude of the spatiotemporal discrepancy multiplied by 0.5. In other words, the sum of both biases would cover the whole magnitude of the spatiotemporal discrepancy (see row 1 in part C of Fig. 1). If the perceived signal relation decreases, the observer would integrate them to a lesser extent. As a result, the magnitudes of the kappa and tau effects decrease if the signals remain equally reliable (see row 4 in part C of Fig. 1). In this case, the sum of the assigned weights would be less than 1 and one would speak of “partial integration”.^2^ The reliability weighting principle basically affects the “symmetry” of both biases. That is, for a given integration strength the precision of each signal determines the relative magnitude of each bias. For example, in case of partial integration (e.g., with the overall strength of 0.8) and a more precise spatial signal (with a weight of 0.6), the kappa effect would amount to 0.6 and the tau effect to 0.2 of the spatiotemporal discrepancy (see row 6 in part C of Fig. 1). Below, more details are provided for how this approach can be applied to the classic paradigm with three stimuli.

Imagine that the two spatial intervals are about 5 cm each and the two temporal intervals are of 0.2- and 0.8-s duration. If the observer decides to fully integrate the signals and trusts both to the same degree (i.e., if the reliability of spatial and temporal signals is equal), the perceived spatial and temporal intervals will be exactly in between what the temporal and spatial signals suggest (Fig. 1, row 1 in part C). More specifically, given a mean velocity of 10 cm/s (v=s/t => [5 cm + 5 cm]/[0.2 s + 0.8 s]), each predicted temporal interval would amount to 0.5 s (t=s/v => 5 cm/10 cm/s). A weighted average of the real (0.2 and 0.8 s) and predicted (0.5 and 0.5 s) temporal intervals would then reveal the values of 0.35 (mean _real & predicted_ = real interval*weight + predicted interval*weight => 0.2*0.5 + 0.5*0.5) and 0.65 s (0.8*0.5 + 0.5*0.5) for the real intervals of 0.2 and 0.8 s, respectively. Thus, the shorter temporal interval would be overestimated and the longer temporal interval would be underestimated consistent with the kappa effects. The same logic can be applied to the perception of spatial intervals and predict the tau effects for the same physical stimulus conditions. In particular, the real spatial intervals (5 cm) will be averaged with the durations derived from the mean velocity (predicted intervals: s=V*t => 10 cm/s*0.2 s = 2 cm; 10 cm/s*0.8 s = 8 cm) and the perceived intervals would amount to 3.5 (mean _real & predicted_ = real interval*weight + predicted interval*weight => 5*0.5 + 2*0.5) and 6.5 cm (5*0.5 + 8*0.5), respectively. Accordingly, the same spatial interval would be under- and overestimated depending on the temporal intervals consistent with the tau effects. A change in the relative signal precision and/or in the magnitude of signal correlation systematically changes the kappa and tau effects (see Fig. 1, part C, rows 2, 3, 4, 5, and 6). If the observer believes that the signals are unrelated, then the signals are not integrated. Accordingly, no kappa and tau effects are expected. By analogy to some observations in the domain of visual-auditory integration (Wallace et al., 2004), reversed kappa and tau effects can occur under these conditions.

The basic algebra behind the constant velocity hypothesis and the reliability weighting rule is the same (weighted sum of individual stimulus information). The latter, however, can be construed as an extension of the former in that it explains where the relative signal weights come from. The reliability weighting rule thus allows us to predict and to test (qualitatively as well as quantitatively) how the kappa and tau effects depend on relative signal precision. Some indirect support for this rule already exists. It has been reported that an increase in the total duration of the stimuli in the classic paradigm decreases the magnitude of the kappa effect and simultaneously increases the magnitude of the tau effect (Huang & Jones, 1982**;** Jones & Huang, 1982). The authors reasoned that “when … judgments are made more difficult, the context – temporal or spatial – is more salient” (p.132 in Jones & Huang, 1982). Such a pattern of results would nicely fit the predictions of the present approach when the authors’ conclusion is correct. An increase in the total stimulus duration, and thus a decrease in mean imputed velocity, could entail a relative increase in the precision of spatial as compared to temporal information.

Several observations made beyond the standard paradigm are also in line with the reliability weighting rule. For example, in a series of experiments, in which the perceived duration and spatial displacement of visually presented lines and dots was measured, the duration judgments were strongly affected by the actual spatial displacement but the displacement judgments were unaffected by the actual stimulus duration (Casasanto & Boroditsky, 2008; see also, e.g., Casasanto et al., 2010, and Merritt et al., 2010, for similar results). Cai and Connell (2015) showed that such a dominance of spatial information is not generally valid. The authors demonstrated an inverse relation between temporal and spatial information (i.e., a dominance of temporal information) when the acuity of the spatial information was reduced by providing the crucial spatial information through a less precise haptic modality (see also Cai & Wang, 2022, for related results).

The unity assumption has not been considered so far to my knowledge in the context of the kappa and tau effects. The mathematics behind this concept are more complex (see, e.g., Shams & Beierholm, 2010), but the reasoning and derived predictions are rather simple. In essence, observer estimates the magnitude of signals’ relation based on a variety of factors, such as their spatio-temporal correlation or task instructions. This estimate then determines the magnitude of integration, i.e., the magnitude of the kappa and tau effects in the present context. Consider for example that researchers usually induce a spatial-temporal discrepancy to quantify the kappa and tau effects in that spatial intervals do not correspond to temporal intervals (e.g., by pairing two different spatial intervals with two equal temporal intervals in the standard paradigm). Consequently, there is a discrepancy between real (spatial or temporal) intervals and those expected according to the imputed uniform stimulus motion (see also Footnote 1). An increase in the magnitude of this discrepancy should decrease the kappa and tau effects according to the unity assumption (see, e.g., Roach et al., 2006, for related findings from audio-visual integration). This is because the inferred (cor-)relation between temporal and spatial signals should decrease.

A similar idea to that I suggest here has already been implemented in a Bayesian model (Cai et al., 2018). This model states that interactions between different cognitive dimensions such as time and space occur because a noisy memory trace of a certain dimension is integrated with the expectation (i.e., prior belief) of a certain magnitude on this dimension. Such expectations are assumed to be learned based on correlations that exist across different dimensions (e.g., it lasts longer to travel a larger distance). This aspect of the model is, in essence, the unity assumption. Moreover, the relative impact of each dimension depends on how noisy its memory is. For example, if memory for temporal aspects of an event is very reliable then little or no impact of the spatial aspects of this event are expected. This is the reliability weighting principle. The validity and scope of such a model remain to be determined due to only a few experiments that directly tested its predictions.

In the present study, I started to examine the multisensory idea. More specifically, I aimed to test the two basic predictions mentioned above. First, I considered how the total duration and the spatial extent of sensory stimulation that determine velocity of stimulus motion presumably imputed by the participants (i.e., “reliability weighting”) could affect the magnitudes of the kappa and tau effects. Second, I tested how the kappa and tau effects could change as a function of spatial-temporal discrepancy (i.e., of “unity assumption”). Three experiments are reported below. In Experiment 1, I focused on the kappa effect and observed results, which were in line with the predictions of the multisensory approach. In Experiment 2, I used a very similar method and aimed to focus on the tau effect. Here I did not observe the tau effect. In Experiment 3, I used exactly the same stimulation for spatial and temporal judgments and replicated the results of Experiments 1 and 2.

## Experiment 1

In Experiment 1, I employed a version of the standard paradigm including three stimuli that appeared at different spatial locations and at different points in time. Two basic parameters were systematically varied. First, the overall duration and spatial extent of stimulation that determine the mean velocity of presumably imputed stimulus motion. Second, the deviation between the temporal and spatial intervals, i.e., the magnitude of spatio-temporal discrepancy.

A decrease in stimulation duration and an increase in spatial extent of stimulation (i.e., an increase in imputed velocity) was expected to increase the impact of spatial information on time perception, i.e., the kappa effect (see alsoHuang & Jones, 1982**;** Jones & Huang, 1982). This can be assumed due to the reliability weighting principle, i.e., because the relative precision of spatial information should increase (or the relative precision of temporal information should decrease) with an increase in stimulus velocity (cf., e.g., row 1 with 3, and row 4 with 6 in panel C of Fig. 1). As mentioned above, the work of Huang and Jones (Huang & Jones, 1982; Jones & Huang, 1982) suggested that the more difficult the primary task is, the stronger the effect of the context. The authors argued that the temporal (spatial) discrimination becomes more difficult with an increase (a decrease) in stimulus velocity. Thus, the effect of spatial (temporal) information on temporal (spatial) judgments, i.e., the kappa (tau) effect should increase (decrease) with velocity. The reliability weighting rule provides a plausible explanation for why this should be so, in line with the reasoning of Huang and Jones. For example, the precision of spatial information might increase with velocity because the memory for spatial locations becomes less demanding. In other words, the spatial information might become less noisy and thus receive more weight if velocity increases.

An increase in spatio-temporal discrepancy was expected to decrease the kappa effect. This prediction is derived from the unity assumption suggesting that the magnitude of sensory integration depends on how strongly the signals are correlated (cf. rows 1, 2, and 3 with rows 4, 5, and 6 in the panel C of Fig. 1). Accordingly, an increase in spatio-temporal discrepancy should entail a decrease in participants’ belief that the spatial and temporal signals relate to the same event (i.e., to the motion of a single stimulus with constant velocity). As a result, they should integrate both signals to a lesser extent, and this should be expressed in a decrease of the kappa effect. A similar pattern of results has been reported for audio-visual integration by Roach et al. (2006). In this study, the integration of auditory and visual signals (indicated by their mutual attraction in perception) decreased and eventually broke down when the multisensory discrepancy increased.

### Methods

#### Participants

Nineteen students of the University of Würzburg were recruited through the participant-acquisition system (SONA systems). They provided informed consent before participation and received course credit for their participation. The sample included 17 females and two males (age: *M* = 21 years, *SD* = 4). The sample size was determined a priori and ensured a power of .80 (*α* = .05) for effect sizes of about *d* = 0.6.

The kappa and the tau effects appeared to be robust phenomena that were usually demonstrated using few observers (see, e.g., Cohen et al., 1953; Helson & King, 1931; Huang & Jones, 1982; Masuda et al., 2011). For example, the effect sizes (*d*) for the kappa effect were between 0.8 and 5.0 in the study of Cohen et al. (1953), which would require between three and 11 participants to demonstrate this effect (given a power of .80 and *α* of .05). Thus, the sample size of Experiment 1 appeared to be appropriate even if a possible uncertainty around the effect size estimate and a possible publication bias are taken into account (cf., Anderson et al., 2017).

The study was conducted in accordance with the ethical guidelines of the German Psychological Society (DGPs) and was approved by the local ethics committee (Ethikkommission des Institutes für Psychologie der Humanwissenschaftlichen Fakultät der Julius-Maximilians-Universität Würzburg, GZEK 2020-88).

#### Apparatus

The experiment was programmed using E-Prime software (Version 3.0; Psychology Software Tools, Pittsburgh, PA, USA) and was run in an online format (using “E-Prime go” application). That is, participants had to download the program files and to perform the experiment on their own computers (running Windows).

#### Trial procedure and stimuli

All stimuli were presented on a gray background. Each trial started with three number signs displayed in light gray side by side (###) for 1,000 ms in the middle of the screen. Then the screen was blank for 1,000 ms. The main stimuli were three black circles (14 pixels in diameter) presented subsequently one by one for 100 ms each. The spatial positions of the circles as well as the temporal intervals between them systematically varied, as described below (see *Design*). The first and the third circles were always equidistant to the center of the screen. The task was to judge whether the temporal interval between the first and the second circles was longer than the interval between the second and the third circles (left mouse button), or vice versa (right mouse button). During initial practice trials, feedback was given about whether the response was correct (German words for “correct” [in green] and “wrong” [in red] were displayed for 250 ms). During the main experiment, no feedback was given.

#### Design

**Fig. 2:**
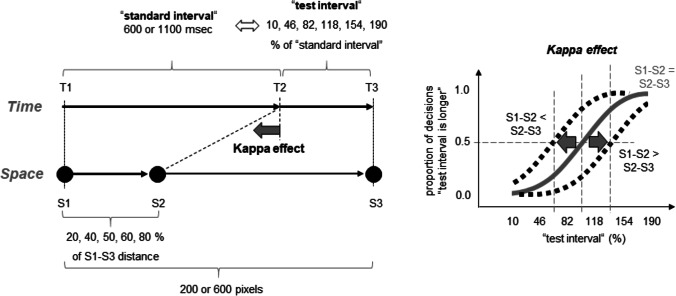
Independent variables (left part) and the measurement of the kappa effect (right part) in Experiment 1. See main text for details

Figure 2 (left part) shows the implemented variations of the temporal and spatial characteristics of the main stimuli (i.e., circles). The spatial positions of the three circles are denoted as S1, S2, and S3. The times of their presentation as T1, T2, and T3. A method of constant stimuli was applied so that one temporal interval (either T1–T2 (50% of trials) or T2–T3 (50% of trials)) served as a “standard stimulus” (“standard interval”), whereas the other interval served as a “test stimulus” (“test interval”; see also Masuda et al., 2011, for a similar method). The standard interval lasted either 600 or 1,100 ms. The duration of the test interval varied between 10% and 190% in equidistant steps of 36% in respect of the duration of the standard interval. This methodical part allowed me to measure participants’ time perception.

In order to examine a possible impact of spatial characteristics on time perception, I varied the spatial position of the second circle (S2) so that the S1–S2 distance amounted to 20, 40, 50, 60, and 80% of S1–S3 distance for each standard and test interval combination. This was an experimental manipulation of the spatial-temporal discrepancy. Hereafter, I refer to this manipulation as if the standard interval was always presented first (as in the example shown in Fig. 2). That is, if I speak of the 20% and 40% conditions, I mean that the spatial interval associated with the temporal standard interval was shorter than that associated with the test interval. Actually, when the test interval was presented first, these conditions (i.e., 20% and 40%) corresponded to an S1–S2 of 60% and 80%, respectively.

The S1–S3 distance was either 200 or 600 pixels. This variation of the spatial extent between S1 and S3 as well as the temporal variation of the standard interval (i.e., 600 or 1,100 ms) served as an experimental manipulation of the mean imputed stimulus velocity and constituted four velocity levels. The slowest velocity was present when the S1–S3 distance was short (200 pixels) and the standard interval was long (1,100 ms). I refer to this condition as V1 hereafter. The fastest velocity condition included a large distance (600 pixels) and a short standard interval (600 ms; V4). The remaining conditions were labeled correspondingly as V2 (200 pixels and 600 ms) and V3 (600 pixels and 1,100 ms). The mean velocities can be approximated here by 0.09 (V1), 0.17 (V2), 0.27 (V3), and 0.5 (V4) pixels/ms, as computed according to:

$$V=\frac{\left({}^{"}S1-S{3}^{"}\right)/2}{{}^{"}{standard\ interval}^{"}}$$

I thus used a 5 (spatial position of second circle (i.e. “spatial-temporal discrepancy”)) × 4 (velocity) × 6 (test interval) design.

The main experiment was divided into two separate sessions. Participants were encouraged to perform the sessions on separate days or at least to take a longer break between them (I did not have the duration of the breaks under control due to an online format of the study). Each session consisted of four blocks of trials including 120 trials each. Participants were encouraged to make breaks after each block. Each combination of spatial and temporal variables was presented in a random order in each block and was repeated eight times (disregarding whether the standard or the test interval was presented first) in the course of the whole experiment. At the beginning of each session, participants performed 12 practice trials, which were randomly chosen from the pool of all experimental trials and in which visual feedback was provided about whether the perceptual judgment was correct or not (see *Procedure and stimuli*). These trials were not included in the analyses.

#### Data analysis

For each test interval, I computed the proportion of trials in which the test interval was judged as longer. This was done for each spatial position of the second circle (i.e., for each level of spatial-temporal discrepancy) and for each velocity condition. I then used a local model-free fitting procedure (Zychaluk & Foster, 2009) to estimate psychometric functions based on the observed proportion data and to determine the so-called points of subjective equality (PSE). The PSE represents a test interval at which the proportion of “test interval is longer” decisions amounted to 50% and indicates how long the standard interval is perceived as compared to the test interval.

The right part of Fig. 2 shows how the kappa effect can be measured using this method. If the second circle appears in the middle between the first and third circle (i.e., in the 50% condition where S1–S2 = S2–S3), then the PSE should approximate the duration of the standard interval (i.e., a test interval of about 100%). If the second circle appears closer to the first circle (i.e., S1–S2 < S2–S3), then the psychometric function should shift to the left. Accordingly, the PSE should decrease. In contrast, if the second circle appears closer to the third circle (i.e., S1–S2 > S2–S3), then the psychometric function should shift to the right and the PSE should increase. Thus, the kappa effect can be visible here as a difference in PSEs across the S1–S2 distance conditions.

To test whether the task in fact becomes more difficult the higher the imputed velocity is, I also looked at the just noticeable difference (JND). This measure was computed by identifying the test intervals corresponding to the 25% and 75% points of the psychometric function and then halving the difference between these values. The larger the value of this measure, the more difficult a task usually is.

#### Transparency and openness

The data have been made publicly available (https://osf.io/ykasw/). This study was not preregistered. The data were collected from October–December 2021.

### Results and discussion

**Fig. 3:**
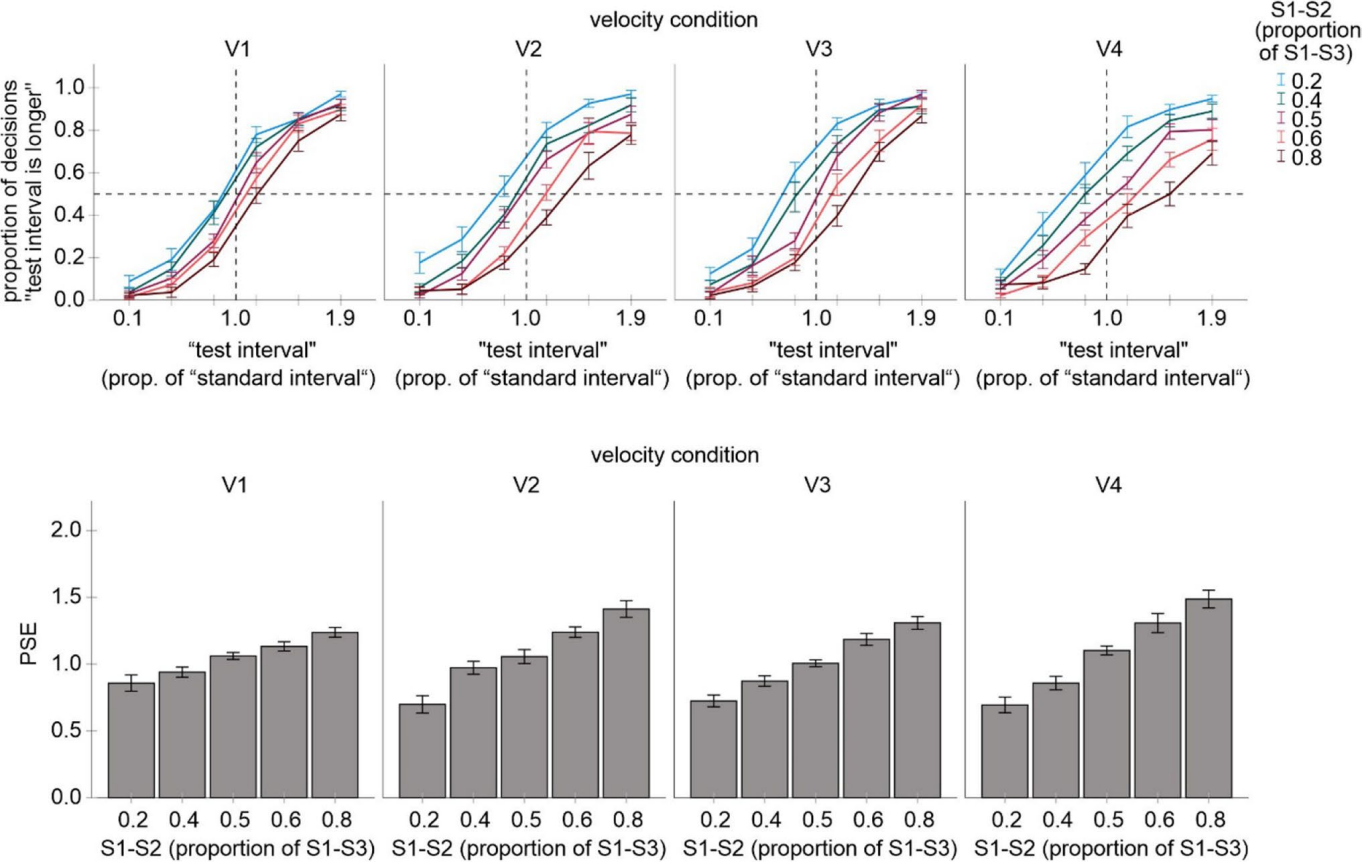
Results of Experiment1. **Upper part:** mean proportion of “test interval is longer” judgments as a function of test interval for each velocity and each spatial distance condition. **Lower part:** mean PSE values for each velocity and each spatial distance condition. Error bars are standard errors. *PSE* point of subjective equality

One participant was excluded from further analyses because of low discrimination performance (mean decision rates were about 0.5 across the test interval, so that PSE estimations were not meaningful). The mean *r*^2^ of this participant (0.25) was more than 3 *SD* below the mean *r*^2^ of the other participants (0.91, *SD* = 0.07). The mean judgment data and the corresponding PSE values of the remaining participants are shown in Fig. 3. As can be seen, the implemented spatio-temporal discrepancy caused systematic biases in the temporal judgments consistent with the kappa effect.

#### PSEs

An ANOVA including the S1–S2 distance and velocity as factors and PSE values as a dependent measure revealed a significant main effect for S1–S2 distance, *F*(4, 64) = 41.32, *p* < .001, *η*_*p*_^*2*^ = .721, a significant main effect for velocity, *F*(3, 48) = 3.17, *p* = .033, *η*_*p*_^*2*^ = .165, and a significant interaction between both factors, *F*(12, 192) = 3.77, *p* < .001, *η*_*p*_^*2*^ = .191. Separate ANOVAs conducted for each velocity condition revealed a significant main effect for S1–S2 distance in each analysis, *F*(4, 64) = 13.46, *p* < .001, *η*_*p*_^*2*^ = .457 (V1), *F*(4, 64) = 22.72, *p* < .001, *η*_*p*_^*2*^ = .587 (V2), *F*(4, 64) = 33.54, *p* < .001, *η*_*p*_^*2*^ = .677 (V3), *F*(4, 64) = 27.16, p < .001, *η*_*p*_^*2*^ = .629 (V4). An increase of the S1–S2 distance entailed an increase of PSEs that tended to be more pronounced the higher the velocity was. This pattern indicated a systematic kappa effect that increased with an increase in spatio-temporal discrepancy and in velocity.^3^

The observed change of the kappa effect with velocity was in accordance with the postulated hypotheses and indicated that the impact of spatial information increased with an increase in velocity. However, a decrease rather than an increase in the kappa effect was expected with an increase in spatio-temporal discrepancy. This result does not necessarily speak against my prediction as the analyses of the absolute kappa values can mask an actual decrease of the kappa effect when this effect is considered with respect to the magnitude of each spatio-temporal discrepancy. In other words, if the kappa effect is considered in proportion to the magnitude of spatio-temporal discrepancy, it can decrease even if its absolute magnitude increases with discrepancy. This would mean that the relative impact of spatial information on temporal perception decreases with discrepancy in line with the postulated hypothesis (even though the absolute kappa increases).

#### Spatial weights

**Fig. 4:**
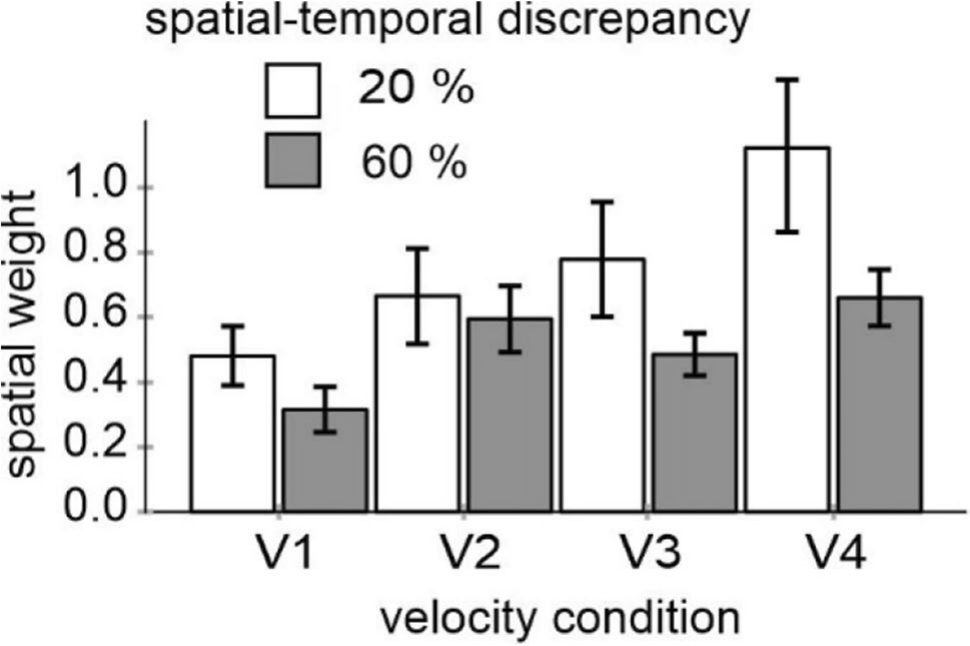
Spatial weights reflecting the influence of spatial information on temporal judgments (0 = no influence; 1 = maximum influence)

Accordingly, I computed an index indicating the relative impact of spatial information, i.e. a “spatial weight.” It reflects how large the influence of spatial information is on a scale between 0 (no influence) and 1 (maximum influence suggesting that temporal judgments were based on spatial information). I first subtracted the PSEs of the 0.5 S1–S2 condition from each other S1–S2 condition. These difference values (i.e., the kappa effects) were then averaged for the 0.2 and 0.8 as well as for the 0.4 and 0.6 conditions (disregarding the sign) and divided by 0.6 and 0.2, respectively (because the spatial deviation of the second circle compared to the 0.5 condition was 60% and 20% respectively). Figure 4 shows these spatial weight values.

An ANOVA including these values as a dependent measure, and velocity (V1, V2, V3, V4) and spatio-temporal discrepancy (20% and 60%) as factors revealed a significant main effect of velocity, *F*(3, 48) = 7.10, *p* < .001, *η*_*p*_^*2*^ = .307, and a significant main effect of discrepancy, *F*(1, 16) = 8.51, *p* = .010, *η*_*p*_^*2*^ = .347. The interaction was not significant, *F*(3, 48) = 1.29, *p* = .288, *η*_*p*_^*2*^ = .075. All weights were significantly different from zero, all *p* < .001, and *d* values ranged between 1.0 and 1.8. Spatial weights increased with velocity, confirming the preceding analysis of PSE values. More importantly here, spatial weights decreased with an increase in the magnitude of spatio-temporal discrepancy.

#### JNDs

An ANOVA including velocity and spatio-temporal discrepancy as factors revealed a significant main effect of velocity, *F*(3, 48) = 11.04, *p* < .001, *η*_*p*_^*2*^ = .408. The main effect of discrepancy and the interaction were not significant, *F*(4, 64) = 1.99, *p* = .107, *η*_*p*_^*2*^ = .111 and *F*(12, 192) = 1.26, *p* = .243, *η*_*p*_^*2*^ = .073 . This outcome indicated an increase in JNDs with an increase in velocity, suggesting that the task became more difficult. Mean values were .32, .35, .31, and .41 for V1, V2, V3, and V4 conditions, respectively, and pairwise comparisons revealed a significant difference between V1 and V4 (see also the slopes of the psychometric functions in Fig. 3, which tend to become flatter with an increase in velocity). This is in line with the prediction that an increase in the kappa effect with velocity is accompanied by an increase in task difficulty (i.e., by a decrease in precision or reliability of temporal compared to spatial information).

Overall, the results supported my hypotheses and indicated that the kappa effect can be understood as an indicator of sensory integration of spatial and temporal information based on the reliability weighting principle and unity assumption.

## Experiment 2

In Experiment 2, I focused on the tau effect using a very similar setup to that in Experiment 1. I again experimentally varied the overall duration and spatial extent of stimulation (i.e., presumably imputed velocity) and the spatio-temporal discrepancy, and examined the consequences of these manipulations on spatial perception. An increase in velocity was expected to decrease the impact of temporal information on spatial perception, i.e. the tau effect (see also Huang & Jones, 1982**;** Jones & Huang, 1982), due to reliability weighting. An increase in spatio-temporal discrepancy was expected to decrease the tau effect due to unity assumption (see also Experiment 1 and *Introduction*).

### Methods

#### Participants

Participants were recruited through the participant pool (SONA systems) of the University of Würzburg. They provided informed consent before participation and received either course credit or monetary compensation for their participation. The sample included 13 females and four males (age: M = 22 years, SD = 4). I aimed to have the same sample size as in Experiment 1 (i.e., n = 18). One participant, however, did not participate despite registration. I expected the effect size for the tau effect to be rather large and comparable to the effect size of the kappa effect as systematic tau effects were also observed using a few participants only (e.g., only three in the study of Huang & Jones, 1982).^4^ Moreover, the results of Experiment 1 confirmed a rather large magnitude of the kappa effect (d values were between 1.0 and 1.8) and its predicted modulations (*η*_*p*_^*2*^ = .307 and *η*_*p*_^*2*^ = .347 for the effects of imputed velocity and spatial-temporal discrepancy; see spatial weights in the *Results and discussion* section of Experiment1), which would require a sample size of between four and eight participants (given a power of .80 and *α* of .05).

#### Apparatus

The apparatus was the same as in Experiment 1.

#### Trial procedure and stimuli

The trial procedure and stimuli were the same as in Experiment 1 except for the task. The task in Experiment 2 was to judge whether the spatial distance between the first and the second circles was larger than the distance between the second and the third circles (left mouse button), or vice versa (right mouse button).

#### Design

**Fig. 5:**
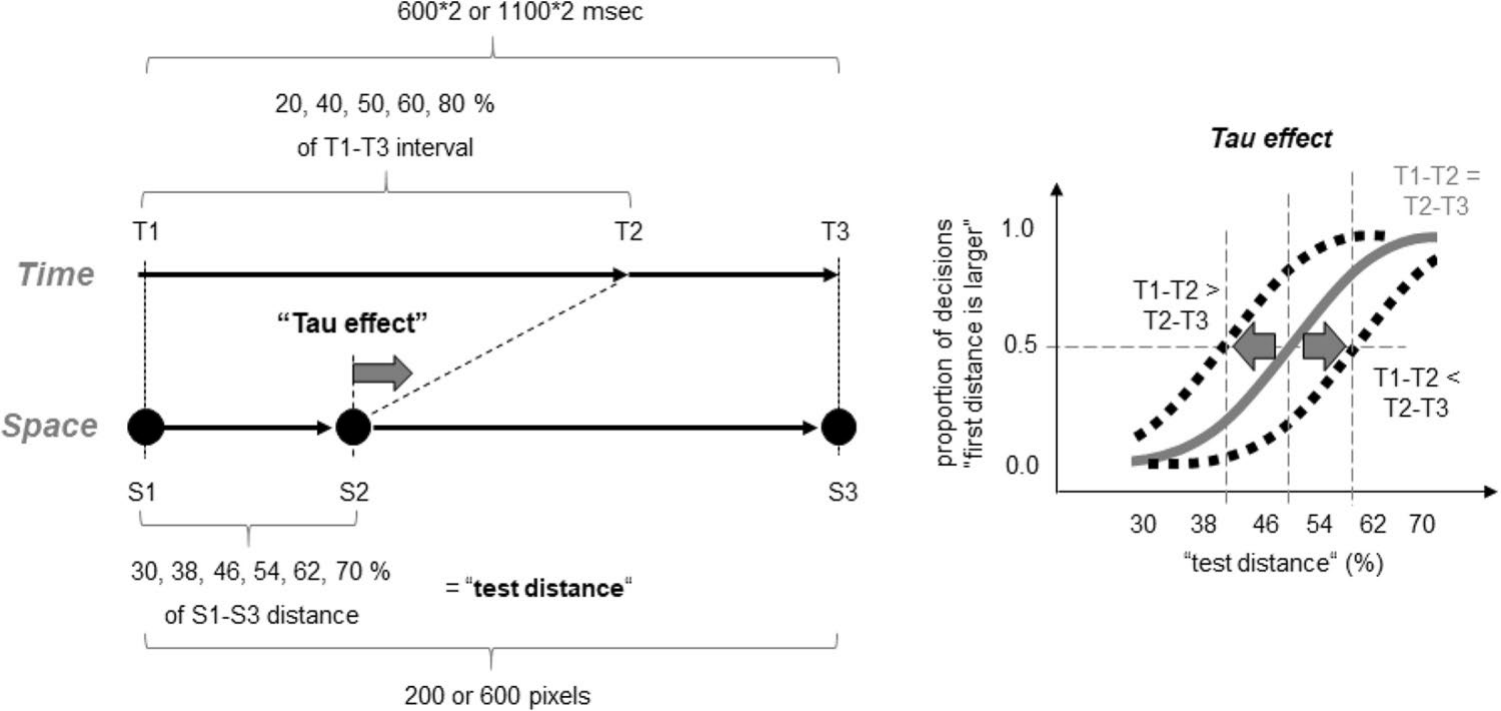
Independent variables (left part) and the measurement of the tau effect (right part) in Experiment 2. See main text for details

With this experiment, I aimed to examine a possible impact of temporal stimulus characteristics on spatial perception. The design of Experiment 1 was thus adjusted accordingly in Experiment 2 (see Fig. 5, left part). I now treated the spatial position of the second circle, i.e., the S1–S2 distance as a “test stimulus” (“test distance”). The S1–S2 distance varied between 30 and 70% in equidistant steps of 8% in respect to the S1–S3 distance (which amounted to either 200 or 600 pixels, as in Experiment 1). This methodical part allowed us to measure participants’ spatial perception.

A spatial-temporal discrepancy was induced by manipulating the T1–T2 interval, which amounted to 20%, 40%, 60%, and 80% of the T1–T3 interval. The T1–T3 interval lasted either 1,200 or 2,200 ms (corresponding to doubled standard intervals of Experiment 1).

The mean velocities of Experiment 2 were comparable to the mean velocities of Experiment 1. They can also be approximated here by 0.09 (V1), 0.17 (V2), 0.27 (V3), and 0.5 (V4) pixels/ms, as computed according to:

$$V=\frac{{}^{"}S1-S{3}^{"}}{{}^{"}T1-T{3}^{"}}$$

Again a 5 (temporal position of second circle (i.e. “spatial-temporal discrepancy”)) × 4 (velocity) × 6 (test distance) design was used. The rest of the design was the same as in Experiment 1.

#### Data analysis

For each test distance, I computed the proportion of trials in which the first spatial distance (i.e., S1–S2) was judged as larger. This was done for each temporal position of the second circle (i.e., for each spatial-temporal discrepancy) and for each velocity condition. I then estimated psychometric functions and determined the PSEs as in Experiment 1.

The right part of Fig. 5 shows how the tau effect can be measured using this method. If the time interval T1–T2 is equal to the time interval T2–T3 (i.e., in the 50% condition), then the PSE should approximate the half of the S1–S3 distance (or about 50% of the test distance). If the second circle appears temporally closer to the third circle (i.e., T1–T2 > T2–T3) then the psychometric function should shift to the left. Accordingly, the PSE should decrease. In contrast, if the second circle appears closer to the first circle (i.e., T1–T2 < T2–T3), then the psychometric function should shift to the right and the PSE should increase. Thus, the tau effect can be measured here as a difference between the 50% and each of the other T1–T2 interval conditions.

To test whether the task in fact becomes more difficult the smaller the imputed velocity is I also looked at the just noticeable difference (JND). This measure was computed in the same way as in Experiment 1.

### Results and discussion

**Fig. 6:**
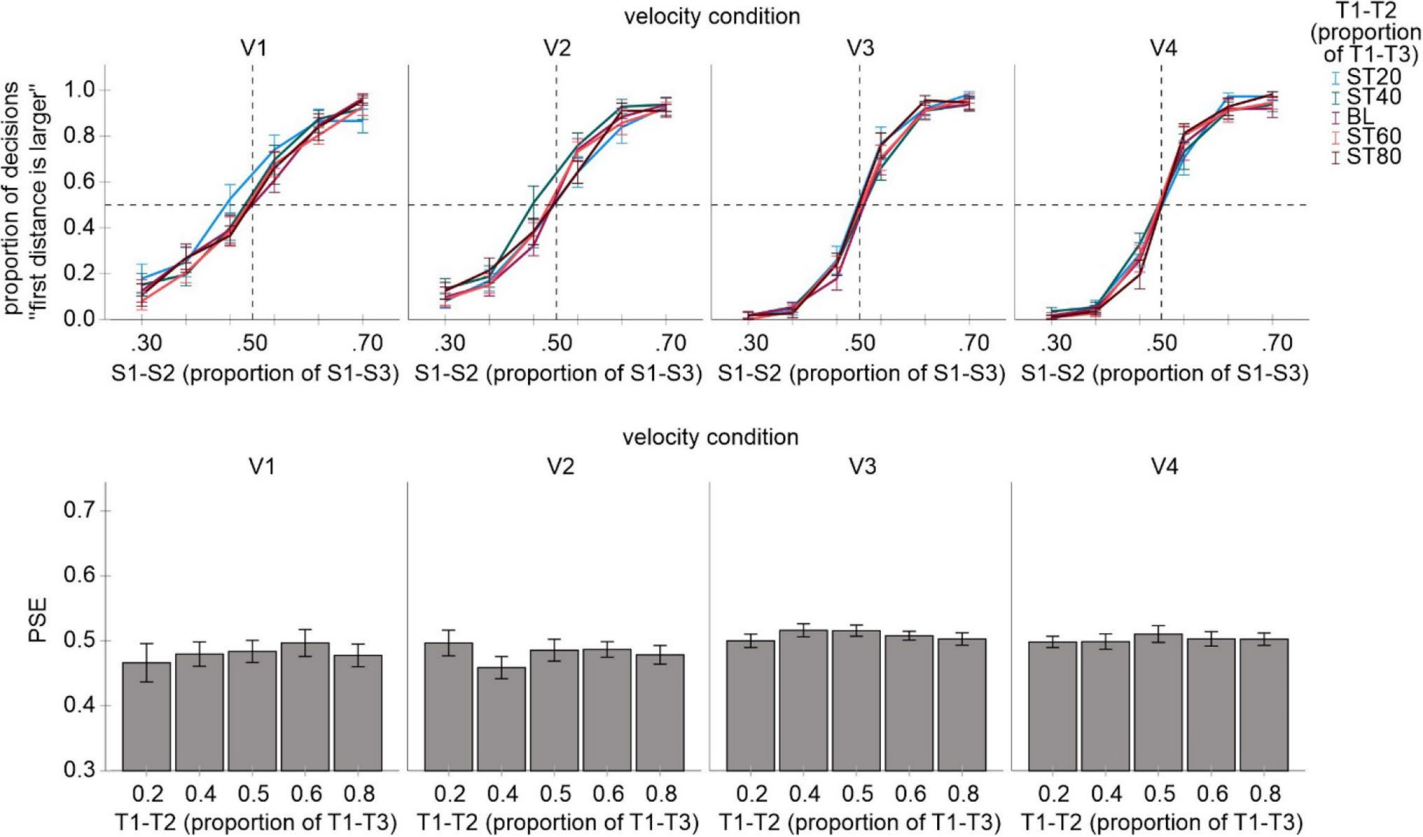
Results of Experiment 2. **Upper part:** mean proportion of “test distance is larger” judgments as a function of test distance for each velocity and each temporal distance condition. **Lower part:** mean PSE values for each velocity and each temporal distance condition. Error bars are standard errors. *PSE* point of subjective equality

Three participants were excluded from analyses due to a very low discrimination performance. Their mean *r*^2^ values (0.57, 0.41 and 0.45) were more than 3 *SD* below the mean *r*^2^ of the other participants (0.92, *SD* = 0.07). The mean data (i.e., proportion rates and the corresponding PSEs) of the other participants are shown in Fig. 6. As can be seen, there was no indication of the tau effect.

#### PSEs

An ANOVA including the T1–T2 interval and velocity as factors and PSE values as a dependent measure did not reveal any significant results, *F*(4, 52) = .64, *p* = .637, *η*_*p*_^*2*^ = .047 (main effect of T1–T2), *F*(3, 39) = 2.22, *p* = .101, *η*_*p*_^*2*^ = .146 (main effect of velocity) and *F*(12, 156) = 1.15, *p* = .321, *η*_*p*_^*2*^ = .082 (interaction). Thus, no systematic influences of temporal stimulus characteristics on spatial judgments were observed under the present conditions.

#### JNDs

An ANOVA including velocity and T1–T2 interval as factors revealed a significant main effect of velocity, *F*(3, 39) = 27.19, *p* < .001, *η*_*p*_^*2*^ = .677. The main effect of T1–T2 interval and the interaction were not significant, *F*(4, 52) = .59, *p* = .673, *η*_*p*_^*2*^ = .043 and *F*(12, 156) = 1.12, *p* = .345, *η*_*p*_^*2*^ = .080. This outcome indicated a decrease in JNDs with an increase in velocity. Mean values were .79, .74, .43, and .38 for V1, V2, V3, and V4 conditions, respectively (see also the slopes of the psychometric functions in Fig. 6, which become steeper with an increase in velocity), suggesting that the task became easier. This is in line with the prediction that an increase in velocity is accompanied by a decrease in task difficulty (i.e., by an increase in precision or reliability of spatial information).

Overall, however, the results did not provide support for the main hypotheses regarding stimulus velocity and spatio-temporal discrepancy as no tau effect was observed.

## Experiment 3

In Experiment 3, I aimed to replicate the results of Experiments 1 and 2. Experiments 1 and 2 were very similar but not identical with respect to physical stimulation. Accordingly, potential reasons for not finding the tau effect in Experiment 2 could, in theory, be related to this difference. I thus aimed to equalize the kappa and tau procedures as far as possible in Experiment 3. For this purpose, I used the same physical stimulation for temporal and spatial judgments. Moreover, I collected these judgments from the same group of participants who did not participate in Experiments 1 and 2. I now expected to see the kappa effect and its modulation depending on imputed velocity and spatio-temporal conflict as in Experiment 1, but no tau effect due to the null result of Experiment 2.

### Methods

#### Participants

Participants were recruited through the participant pool (SONA systems) of the University of Würzburg. They provided informed consent before participation and received either course credit or monetary compensation for their participation. The sample included 13 females and four males (age: M = 26 years, SD = 7). I aimed to have a similar sample size to that in Experiments 1 and 2 (see Experiments 1 and 2 for the justification of the chosen sample size).

#### Apparatus

The apparatus was the same as in Experiments 1 and 2.

#### Trial procedure and stimuli

The trial procedure and stimuli were largely the same as in Experiments 1 and 2. The only difference was that in Experiment 3, spatial and temporal judgments were collected from the same participants in different blocks of trials.

#### Design

**Fig. 7:**
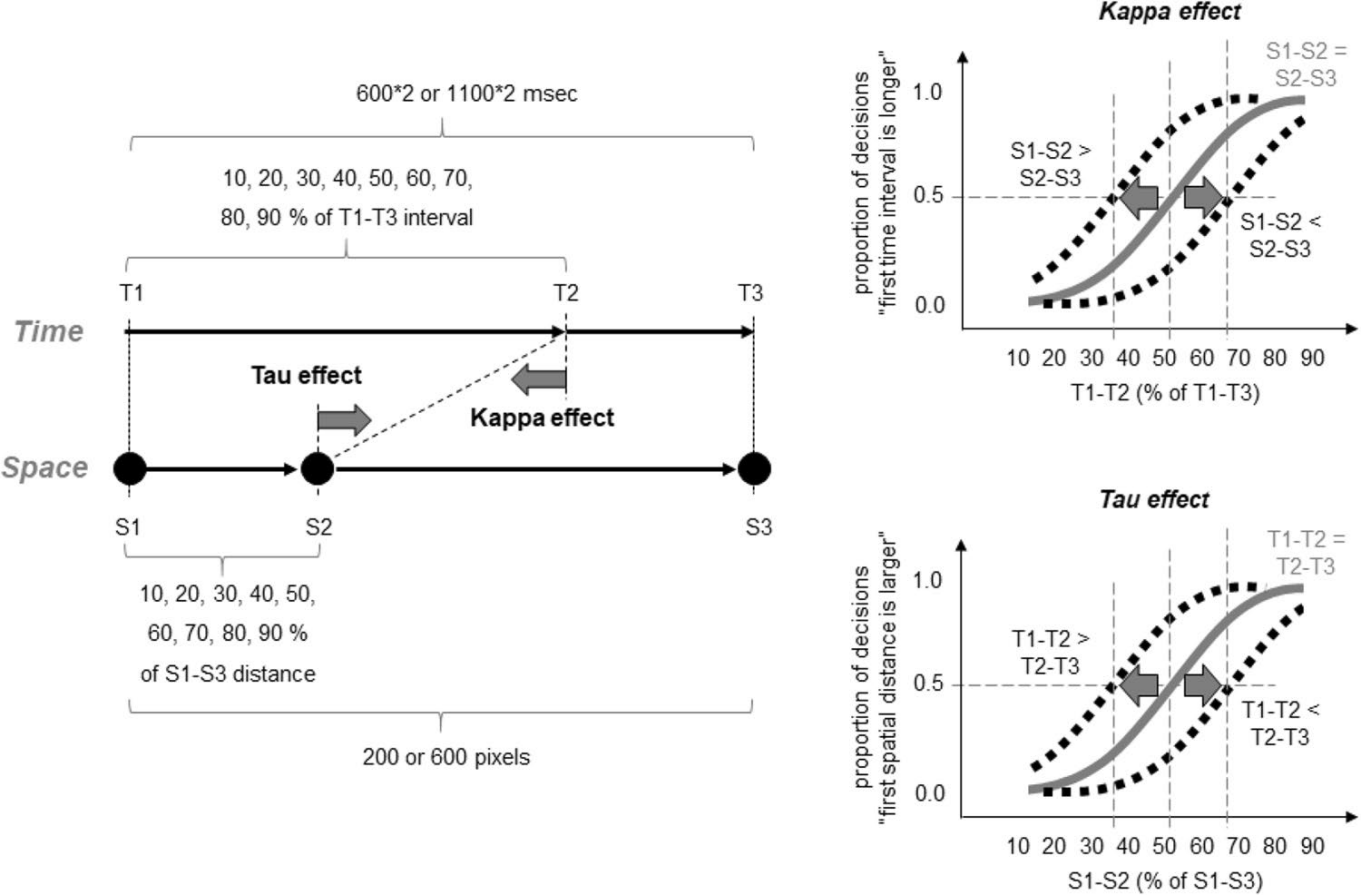
Independent variables (left part) and the measurement of the kappa and tau effects (right part) in Experiment 2. See main text for details

With this experiment, I aimed to examine a possible mutual perceptual attraction between temporal and spatial stimulus characteristics in a single experiment under identical physical conditions. The design of Experiment 3 was thus adjusted accordingly (see Fig. 7, left part). I now treated the spatial position of the second circle (i.e., S1–S2 interval) as a “test distance” when spatial judgments were required and, simultaneously, as a factor that potentially influences time perception when temporal judgments were required. In a similar vein, I treated the temporal position of the second circle (i.e., T1–T2 interval) as a “test interval” when temporal judgments were required and, simultaneously, as a factor that potentially influences space perception when spatial judgments were required.

The S1–S2 distance now varied between 10% and 90% in equidistant steps of 10% with respect to the S1–S3 distance (that amounted to either 200 or 600 pixels as in Experiments 1 and 2). The T1–T2 interval varied also between 10% and 90% in equidistant steps of 10% with respect to the T1–T3 interval (that amounted to either 1,200 or 2,200 ms as in Experiment 2).

In this experiment, I used only two velocity conditions by combining a small S1–S3 distance (200 pixels) with a long T1–T3 interval (2,200; V1) and a large distance (600 pixels) with a short interval (1,200 ms; V4). The mean velocities in these conditions were the same as in Experiments 1 and 2.

Here, I thus used a 9 (temporal or spatial position of second circle (i.e., “spatial-temporal discrepancy”)) × 2 (velocity) × 9 (test interval) design for each judgment type.

The main experiment was divided into four separate sessions. Participants were encouraged to perform the sessions on separate days or at least to take longer breaks between them. Each session consisted of eight blocks of trials including 81 trials each. In one half of the blocks, temporal judgments were required as in Experiment 1. In the other half of the blocks, spatial judgments were required as in Experiment 2. The succession of the block types was random. Participants were encouraged to make breaks after each block. The spatial and temporal variables were presented in a random order in each block. Each combination of these variables (i.e., of S1–S2 distance, T1–T2 interval, and velocity condition) was repeated eight times in the course of the whole experiment. At the beginning of each session, participants performed 20 practice trials (10 spatial judgment + 10 temporal judgments). In these trials, visual feedback was provided about whether the perceptual judgment was correct or not. These trials were not included in the analyses.

#### Data analysis

Data analysis was performed in a similar way as in Experiments 1 and 2. That is, for spatial judgment blocks, I computed the proportion of trials in which the first spatial distance (i.e., S1–S2) was judged as larger as a function of S1–S2 distance. This was done for each temporal position of the second circle (i.e., for each T1–T2 interval) and for each velocity condition. By analogy, the proportion of trials in which the T1–T2 interval was judged as longer was computed for the temporal judgment blocks. This was done for each S1–S2 distance and each velocity condition. I then estimated psychometric functions and determined the PSEs as in the previous experiments.

The right part of Fig. 7 shows how the kappa and the tau effects can be measured using this method. In case of the tau effect, the logic is the same as in Experiment 2. That is, if the T1–T2 interval is equal to the T2–T3 interval, then the PSE should approximate half of the S1–S3 distance. If the T1–T2 interval is larger (smaller) than the T2–T3 then the psychometric function should shift to the left (right). An inverted logic can be applied to the kappa effect. That is, if the S1–S2 distance is equal to the S2–S3 distance, then the PSE should approximate the half of the T1–T3 interval. If the S1–S2 distance is larger (smaller) than the S2–S3 distance then the psychometric function should shift to the left (right). Thus, the kappa and tau effects can be visible here as a difference in PSEs across the S1–S2 and T1–T2 conditions.

### Results and discussion

**Fig. 8:**
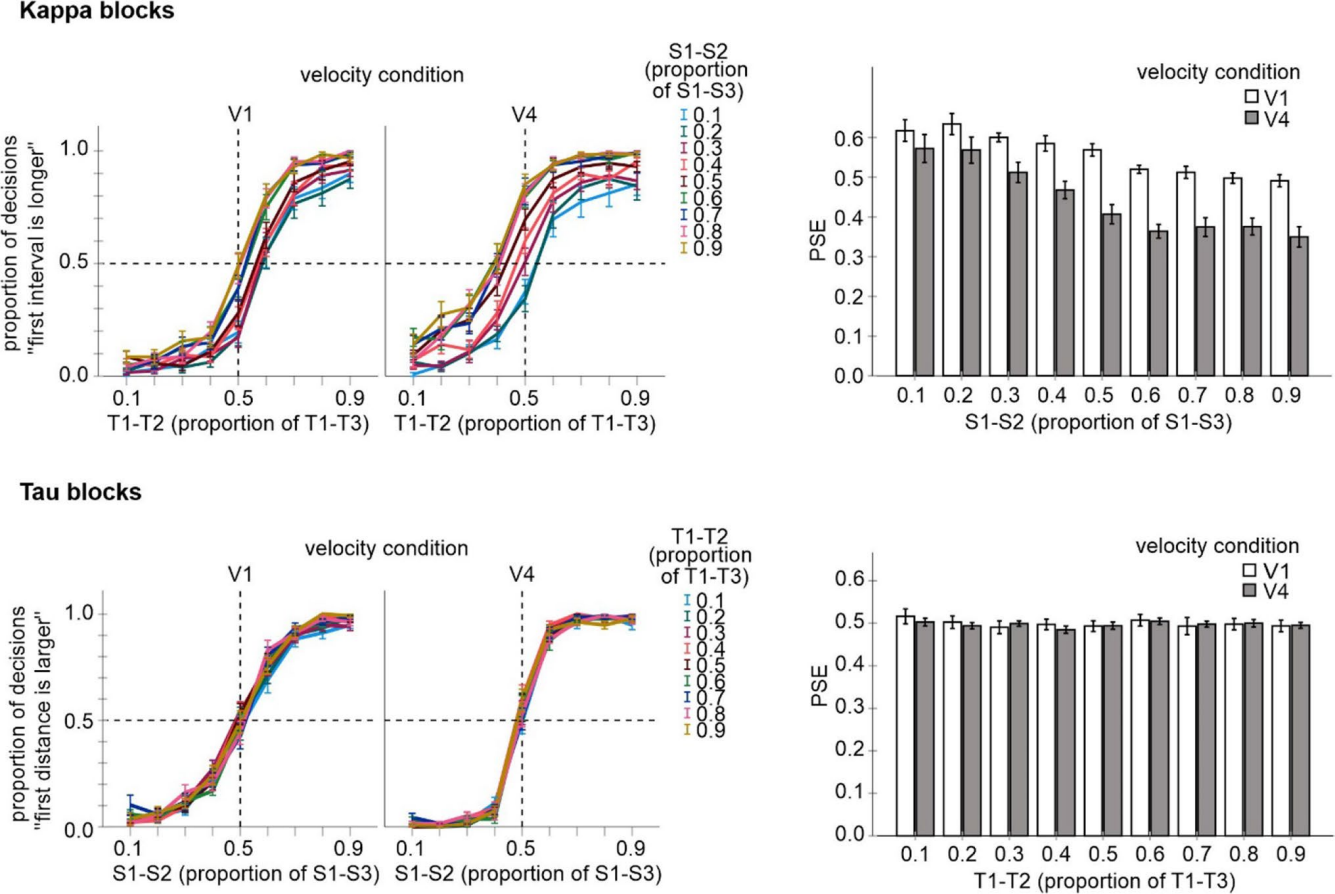
Results of Experiment 3. **Left part:** mean proportion of “first interval is longer” or “first distance is larger” judgments as a function of T1–T2 interval or S1–S2 distance for each velocity and each S1–S2 distance or T1-T2 interval condition. **Right part:** mean PSE values for each velocity and each S1–S2 distance or T1–T2 interval condition. Error bars are standard errors. *PSE* point of subjective equality

The data of one participant from the kappa blocks were excluded from further analyses because of low discrimination performance. The mean *r*^2^ of this participant (0.54) was more than 3 *SD* below the mean *r*^2^ of the other participants (0.92, *SD* = 0.06). Figure 8 shows mean judgment data and the corresponding PSE values for the temporal and spatial judgments (i.e., for the kappa and tau blocks, respectively). As can be seen, the implemented spatio-temporal discrepancy influenced the temporal judgments as in Experiment 1, but not the spatial judgments as in Experiment 2.

#### PSEs and spatial weights in kappa blocks

An ANOVA including the S1–S2 distance and velocity as factors and PSE values of kappa blocks as a dependent variable revealed significant main effects for both factors, *F*(8, 120) = 17.15, *p* < .001, *η*_*p*_^*2*^ = .533, and *F*(1, 15) = 86.62, *p* < .001, *η*_*p*_^*2*^ = .852, and a significant interaction, *F*(8, 120) = 5.06, *p* < .001, *η*_*p*_^*2*^ = .252. Separate ANOVAs conducted for each velocity condition revealed a significant main effect for S1–S2 distance, *F*(8, 120) = 12.87, *p* < .001, *η*_*p*_^*2*^ = .462 (V1), and *F*(8, 120) = 15.23, *p* < .001, *η*_*p*_^*2*^ = .504 (V4). An increase of the S1–S2 distance entailed a decrease of PSE that was stronger pronounced in the higher velocity condition. This pattern indicated a systematic influence of spatial information on temporal judgments (i.e., a kappa effect) being larger for the larger (imputed) stimulus velocity.

To assess whether the magnitude of the assumed spatio-temporal integration varies depending on the magnitude of spatio-temporal discrepancy I again computed an index indicating the relative impact of spatial information, i.e., a “spatial weight” in an analogous way as in Experiment 1. I first subtracted the PSEs of the 0.5 S1–S2 condition from each other S1–S2 condition. These difference values (i.e., the kappa effects) were then divided by 0.2 (0.4 and 0.6 conditions), 0.4 (0.3 and 0.7 conditions), 0.6 (0.2 and 0.8 conditions), and 0.8 (0.1 and 0.9 conditions) and averaged (disregarding the sign) for the 0.4 and 0.6, 0.3, and 0.7, 0.2 and 0.8, 0.1, and 0.9 conditions.

**Fig. 9:**
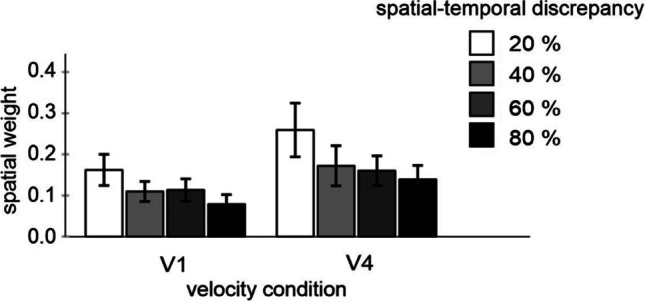
Spatial weights reflecting the influence of spatial information on temporal judgments (0 = no influence; 1 = maximum influence)

An ANOVA including these spatial weights as a dependent measure, and velocity and spatio-temporal discrepancy as factors, revealed a significant main effect of velocity, *F*(1, 15) = 5.33, *p* = .036, *η*_*p*_^*2*^ = .262, and more importantly here, a significant main effect of discrepancy, *F*(3, 45) = 6.88, *p* < .001, *η*_*p*_^*2*^ = .315. The interaction was not significant, *F*(3, 45) = .47, *p* = .705, *η*_*p*_^*2*^ = .030. All weights were significantly different from zero (all *p* < .004). Spatial weights increased with velocity, confirming the preceding analysis of PSE values. More importantly here, spatial weights decreased with an increase in the magnitude of spatio-temporal discrepancy (see Fig. 9 for mean values).

Overall, the spatial weights were substantially smaller in Experiment 3 than in Experiment 1, indicating a smaller impact of spatial information on temporal judgments in Experiment 3. This side effect is likely related to methodical differences between both experiments. In particular, Experiment 3 contained more levels and larger magnitudes of spatio-temporal discrepancies than Experiment 1. That is, on average participants experienced more and larger spatio-temporal discrepancies in Experiment 3 than in Experiment 1. This could lead to an overall decrease of spatio-temporal integration and thus to smaller Kappa effects (according to the unity assumption, see also *Introduction* and Experiment 1).

#### PSEs in tau blocks

As in Experiment 2, the temporal information did not affect the spatial judgments (i.e., no tau effect was observed). An ANOVA including the T1–T2 interval and velocity as factors and PSE values as a dependent measure did not reveal significant effects, *F*(8, 128) = .89, *p* = .528, *η*_*p*_^*2*^ = .053 (main effect T1–T2), *F*(1, 16) = .040, *p* = .845, *η*_*p*_^*2*^ = .002 (main effect velocity), *F*(8, 128) = .432, *p* = .900, *η*_*p*_^*2*^ = .026 (interaction).

#### JNDs in tau and kappa blocks

JNDs decreased with velocity in the tau blocks as in Experiment 2, *F*(1,16) = 114.46, *p* < .001, *η*_*p*_^*2*^ = .877 for the main effect of velocity. Mean values were .098 and .046 for V1 and V4, respectively. The main effect of T1–T2 interval and the interaction were not significant, *F*(8, 128) = 1.20, *p* = .304, *η*_*p*_^*2*^ = .070 and *F*(8, 128) = 1.57, *p* = .139, *η*_*p*_^*2*^ = .090. In the kappa blocks, an analogous analysis revealed a significant main effect of velocity, *F*(1, 15) = 5.51, *p* = .033, *η*_*p*_^*2*^ = .269. As in Experiment 1, an increase in velocity was associated with an increase in JNDs. Mean values were .098 (V1) and .115 (V4). The main effect of S1–S2 distance and the interaction were not significant, *F*(8, 120) = 1.76, *p* = .092, *η*_*p*_^*2*^ = .105 and *F*(8, 120) = .91, *p* = .514, *η*_*p*_^*2*^ = .057, respectively.

Overall, the main results of Experiment 3 replicated and confirmed the results of Experiments 1 and 2 in that a kappa effect was observed that varied with imputed velocity and the magnitude of spatio-temporal discrepancy, and in that no tau effect was observed under very similar task conditions.

## General discussion

The kappa and tau effects are well-known examples of the mutual attraction between spatial and temporal signals in perception. Their origin is not well understood yet. Here I suggest that these effects arise if the system integrates correlated but somewhat discrepant signals relating to a common event based on basic principles of multisensory integration. The present study including three experiments aimed to provide an initial test for this idea. I varied the duration and spatial extent of sensory stimulation that constitute velocity of stimulus motion presumably imputed by the observer to the static displays as well as the magnitude of spatio-temporal discrepancy. The observed results were in line with the predictions of the multisensory approach when time perception was measured (i.e., for the kappa effect). However, no tau effect was observed.

In theory, a lack of the tau effect could mean that the weights assigned to the spatial information during spatio-temporal integration are substantially larger than those assigned to the temporal information under the present conditions. In other words, the temporal and spatial signals were integrated but the temporal signal was too noisy to produce a systematic influence in spatial perception under the present conditions (see also Cai et al., 2018).

On the other hand, a lack of the tau effect could also indicate that temporal and spatial signals were not integrated at all when spatial judgments were required. This could have to do with how attention was distributed across the spatial and temporal aspects of the task. For example, temporal signals could generally be less salient than spatial signals, and thus could more easily be ignored (cf., e.g., Welch & Warren, 1980; Werkhoven et al., 2009). This could result in a strong focus on spatial information. Such a strong focus on one sensory signal should entail a lower magnitude of intersensory integration as compared to conditions in which attention is shared between different signals to a larger degree (see, e.g., Talsma et al., 2007; Werkhoven et al., 2009), possibly because the unity assumption is weakened (Badde et al., 2020). It is also conceivable that the decision to integrate or not to integrate the spatial and temporal information is directly related to how salient the attended signal is. Attending a less salient signal could force the decision to integrate stronger as compared to attending a more salient signal.

Although the present results provided only partial support for the multisensory approach that I put forward, I still believe that this approach is very promising and can enable a better understanding of many related phenomena. For example, it is not limited to the standard kappa and tau paradigm using three stimuli. It can also be applied when the to-be judged intervals are defined by two successive stimuli (e.g., Chen et al., 2016). In this case, the temporal and spatial expectations in a given trial could be based on mean velocity estimates derived from preceding trials. Moreover, the general idea behind this approach does not depend on whether the observer relies on a velocity estimation and is thus applicable to kappa- and tau-like observations where no real or apparent motion is present in the stimulus pattern (e.g., Experiment 6 in Casasanto & Boroditsky, 2008). Here, the temporal and spatial expectations can be derived from learning that, for example, an increase in magnitude on a temporal dimension is often associated with an increase in magnitude on a spatial dimension (e.g., Cai et al., 2018). The suggested idea as well as related models (Cai et al., 2018) can thus potentially explain a wide range of cross-dimensional interactions concerning the magnitude of stimuli based on the relative precision of the signals and their correlation. More research is, however, needed to better evaluate the scope of such an approach and its limitations.

To sum up, the results of the present study suggest that under similar stimulation conditions the impact of spatial information on temporal perception (i.e., the kappa effect) is stronger than the impact of temporal information on spatial perception (i.e., the tau effect). In addition, the kappa effect increases when velocity imputed to the stimuli increases. Also, the relative magnitude of the kappa effect increases when the conflict between the expected and the real temporal information decreases. These results indicate that basic principles of multisensory integration are at work during the emergence of the kappa and the tau effects.

## Footnotes

1 The term “discrepancy” denotes that two signals that relate to a common external event provide deviating information about this event. In experiments on visual-haptic integration, for example, participants grasp a visual object but the visual size of the object does not fully correspond to the magnitude of the hand opening. In typical kappa and tau experiments with three stimuli, the intersensory discrepancy relates to systematic manipulations of one dimension (e.g., spatial) while holding the other dimension (e.g., temporal) constant. Assume that each of two temporal intervals is of the same duration and the spatial intervals are different. Accordingly, there is a difference or discrepancy between these real spatial and temporal intervals and those predicted or expected based on the imputed overall (constant) stimulus velocity. This difference can be called an “intersensory discrepancy”.

2 This type of sensory coupling is often observed in the multisensory domain (e.g., Kirsch & Kunde, 2022).

3 I also ran an ANOVA including S1–S2 distance, standard interval (600, 1,100 ms) and S1–S3 distance (200, 600 px) as factors. This analysis revealed a significant main effect for S1–S2 distance, *F*(4, 64) = 41.32, *p* < .001, *η*_*p*_^*2*^ = .721, and a significant main effect for standard interval, *F*(1, 16) = 7.72, *p* = .013, *η*_*p*_^*2*^ = .325. The PSE increased with S1–S2 distance (see also Fig.3), and was on average slightly larger for the shorter standard interval (M_600_ = 1.08, M_1100_ = 1.03). More importantly, significant interactions were observed between S1–S2 distance and standard interval, *F*(4, 64) = 6.45, *p* > .001, *η*_*p*_^*2*^ = .287, and between the S1–S2 distance and S1–S3 distance, *F*(4, 64) = 2.99, *p* = .025, *η*_*p*_^*2*^ = .157 (all other *p* > .3). The increase of PSE with S1–S2 distance was more pronounced for the shorter standard interval and for the larger S1–S3 distance. These results suggested that the Kappa effect increased when the overall stimulus duration decreased or the overall distance increased, i.e. when velocity increased. This is consistent with the results of the main analyses and confirmed my treatment of the standard interval and S1–S3 distance as a common velocity factor.

4 It was not possible to more exactly estimate an effect size for the tau effect based on previous studies due to a lack of required information.
